# Long-Term Improvement in Cardiorespiratory Fitness Ameliorates Insulin Sensitivity beyond Changes in Visceral/Ectopic Fat among Men with Visceral Obesity

**DOI:** 10.3390/nu16091377

**Published:** 2024-05-01

**Authors:** Adrien Murphy-Després, Dominic J. Chartrand, Isabelle Lemieux, Angelo Tremblay, Jean Bergeron, Paul Poirier, Natalie Alméras, Jean-Pierre Després

**Affiliations:** 1 Centre de Recherche de l’Institut Universitaire de Cardiologie et de Pneumologie de Québec—Université Laval, Québec, QC G1V 4G5, Canada; adrien.murphy-despres.1@ulaval.ca (A.M.-D.); dominic.chartrand@criucpq.ulaval.ca (D.J.C.); isabelle.lemieux@criucpq.ulaval.ca (I.L.); angelo.tremblay@kin.ulaval.ca (A.T.); paul.poirier@criucpq.ulaval.ca (P.P.); natalie.almeras@criucpq.ulaval.ca (N.A.); 2 Department of Kinesiology, Faculty of Medicine, Université Laval, Québec, QC G1V 0A6, Canada; 3 Department of Molecular Biology, Medical Biochemistry, and Pathology, Faculty of Medicine, Université Laval, Québec, QC G1V 0A6, Canada; jean.bergeron@crchudequebec.ulaval.ca; 4 Centre de Recherche du CHU de Québec—Université Laval, Québec, QC G1V 4G2, Canada; 5 Faculty of Pharmacy, Université Laval, Québec, QC G1V 0A6, Canada; 6 VITAM—Centre de Recherche en Santé Durable, CIUSSS de la Capitale-Nationale, Québec, QC G1J 2G1, Canada

**Keywords:** cardiorespiratory fitness, insulin sensitivity, intramuscular fat, lifestyle intervention, visceral obesity

## Abstract

The SYNERGIE study documented the effects on cardiometabolic risk (CMR) indices of a 1-year lifestyle intervention targeting physical activity (PA) and diet followed by a 2-year maintenance period in men with visceral obesity. Improvements in CMR markers and a decrease in low-attenuation muscle (LAM) area were observed after 1 year. Despite a rebound in visceral adipose tissue (VAT) during the maintenance period, insulin resistance (IR) improved. We tested the hypothesis that variations in cardiorespiratory fitness (CRF) and LAM could explain the long-term improvement in IR. A health (*n* = 88; mean age 49.0 ± 8.2 years) and fitness (*n* = 72) evaluation was performed at 0, 1, and 3 years. Participants were classified into two groups based on their CRF response over the maintenance period (worsening: CRF− vs. maintenance/improvement: CRF+). During the maintenance period, changes in the psoas and core LAM areas correlated with changes in IR (*r* = 0.27; *p* < 0.05 and *r* = 0.34; *p* < 0.005) and changes in CRF (*r* = −0.31; *p* < 0.01 and *r* = −0.30; *p* < 0.05). IR improved in the CRF+ group (*p* < 0.05) but remained stable in the CRF− group. Men in the CRF+ group regained half of the changes in VAT volume and LAM at the psoas and mid-thigh compared to the CRF− group (*p* < 0.05). These results support the importance of targeting VAT and CRF/PA for the long-term management of CMR in men with visceral obesity.

## 1. Introduction

In 2010, the American Heart Association introduced the concept of ideal cardiovascular health to emphasize the importance of healthy behaviors (regular physical activity [PA], high-quality diet, nonsmoking, healthy weight) in addition to maintaining normal levels of traditional risk factors (blood glucose, blood cholesterol, and blood pressure) to reduce the burden of cardiovascular disease [1]. As a self-reported level of PA is associated with potential over or under-reporting and misclassification of related risk [2], cardiorespiratory fitness (CRF), an objective physiological consequence of regular participation in moderate- to vigorous-intensity physical activity (MVPA), is a powerful variable to discriminate cardiovascular disease risk [3]. Indeed, a low level of CRF is a stronger predictor of mortality than traditional risk factors such as smoking, hypertension, high cholesterol, type 2 diabetes (T2D), and obesity defined by body mass index (BMI) [3,4]. Thus, maintaining or increasing CRF through regular PA is essential to optimize cardiovascular health, especially for individuals with visceral obesity, as it reduces the risk of developing T2D as well as the related mortality risk [5,6].

Although obesity is considered a risk factor for many cardiometabolic diseases [7], it has been shown that BMI is not an appropriate anthropometric index to assess individual differences in body fat distribution [8]. For instance, the ability of adipose tissue to expand to store excess calories associated with a sedentary lifestyle, physical inactivity, and unhealthy diets is characterized by a substantial individual variation [9]. Visceral obesity is recognized as the form of overweight/obesity associated with a cluster of metabolic disorders [10] and is also partly mediated by an increased accumulation of lipids in normally lean tissues (epi/pericardial fat, liver fat, intramuscular fat [IMF]), a process referred to as ectopic fat deposition. Whereas visceral obesity and excess ectopic fat deposition are known to increase cardiometabolic risk (CMR), high levels of increased IMF have been specifically associated with insulin resistance even after adjusting for BMI, suggesting that this ectopic fat depot and its lipid intermediates, such as diacylglycerols and ceramides, may also contribute to the development of T2D [11,12,13].

Lifestyle interventions targeting PA and nutrition have proven to be effective in the short-term to lower IMF and improve insulin sensitivity in patients with T2D or with obesity [14,15]. To the best of our knowledge, no study has investigated the long-term impact of a lifestyle intervention on IMF and its association with CMR. We had previously reported that a 1-year lifestyle intervention lowered IMF, increased CRF, and improved insulin sensitivity in men with visceral obesity [16]. However, following an additional 2-year maintenance period, men involved in this previous study regained visceral adipose tissue (VAT) despite the fact that their indices of insulin sensitivity were further improved [17], a result deemed counter-intuitive given the associations between VAT and insulin resistance.

Therefore, the purpose of this secondary analysis was to better understand how the 2-year adherence to lifestyle changes after the 1-year intervention period could explain the maintenance or even further improvement in insulin sensitivity despite a rebound in VAT. Could CRF and IMF explain the apparent paradox of improved insulin sensitivity despite a regain in VAT? We tested the hypothesis that the maintenance of CRF through sustained PA habits after the 1-year lifestyle intervention could be an important factor contributing to the maintenance/improvement in insulin sensitivity observed during the 2-year follow-up period.

## 2. Materials and Methods

### 2.1. Subjects

Physically inactive Caucasian men with visceral obesity were recruited (*n* = 144) to participate in a 3-year lifestyle modification program aimed at increasing their level of PA and improving their nutritional habits (the SYNERGIE study). Participants were included in this study if they had a waist circumference ≥90 cm with plasma triglyceride concentrations ≥1.69 mmol/L and/or high-density lipoprotein (HDL) cholesterol levels <1.03 mmol/L, and if they reported less than 30 min of continuous and vigorous PA per week. Participants with a diagnosed T2D, a BMI ≤25 or ≥40 kg/m^2^, or taking medication targeting glucose, lipid metabolism, or blood pressure were excluded. These criteria were selected based on the previous literature [18,19,20,21]. This study was approved by the Ethics Committee of Université Laval under the reference number 2003-242, 1080 (February 2004) and the Ethics Committee of Institut universitaire de cardiologie et de pneumologie de Québec—Université Laval under the reference number 2005-977 (June 2004).

### 2.2. Lifestyle Intervention

The first part of this study was a 1-year lifestyle intervention designed to generate a moderate energy deficit (~500 kcal/day) taking into account mean energy intake and energy expenditure. The intervention was followed by a 2-year period in which participants were asked to maintain their newly adopted healthy habits and their reduced body weight. Participants met with a kinesiologist and a registered nutritionist every 2 weeks for the first 4 months, followed by monthly appointments for the remainder of the 1-year intervention. During the 2-year maintenance period, participants were offered a consultation with a kinesiologist and a nutritionist every 5 to 6 weeks.

### 2.3. Nutritional Counseling

Participants were given tools to help them modify their food habits (e.g., increases in vegetables and fruits, fishes and lean meat sources, low-fat dairy products, and whole-grain products; and reductions in saturated fat, red meat, processed and energy-dense food, such as pastries, fried food, etc.). The registered nutritionists aimed at achieving a macronutrient distribution of 45–50% carbohydrates, 20–25% proteins, and 25–30% lipids.

### 2.4. Physical Activity Counseling

The individualized PA program was based on the participants’ preferences (type of PA, indoor/outdoor, etc.) and on the results of the graded sub-maximal treadmill test to reach the weekly target of 160 min of moderate-intensity endurance exercise. Participants had free access to our fitness center for the first 4 months but were also encouraged to perform PA outside our facilities. After this initial 4-month period, participants could use the setting of their choice to perform their PA, as one of the goals of the study was to empower them regarding their lifestyle habits. A pedometer (SW200 models; StepsCount, Deep River, ON, Canada) was also given to participants as they were also encouraged to reach 10,000 steps per day to optimize their weekly PA level.

This present article focuses on participants who completed the 1-year intervention as well as the 2-year maintenance period and who have been subjected to the computed tomography (CT) scan after the 2-year maintenance period (*n* = 88). Participants had a comprehensive evaluation at baseline, year 1 (after the intervention), and at year 3 (after the 2-year maintenance period). A less exhaustive evaluation was also performed after the first year of the maintenance period (year 2).

### 2.5. Anthropometric Measurements

Height, weight [22], and waist circumference [23] were measured using standardized procedures. Briefly, for weight, height, and waist circumference measurements, subjects were in a standing position and were requested to wear light clothes and be barefoot. Waist circumference was obtained at the top of the iliac crest at the end of a normal expiration, and the mean of two measures within 1.0 cm was used. Weight was measured to the nearest 0.1 kg, and height and waist circumference measurements to the nearest millimeter. BMI was calculated.

### 2.6. Computed Tomography

VAT and subcutaneous adipose tissue (SAT) cross-sectional areas and IMF were assessed by CT at the L2/L3 and L4/L5 intervertebral spaces as well as at the mid-thigh level at baseline, after year 1, and after year 3. CT scans were segmented using the specialized software sliceOmatic (version 4.9—Rev 9b, Tomovision, Montréal, QC, Canada). Partial volumes of VAT and SAT were also calculated using the previously described procedures [24]. Trunk muscles on the L4/L5 scan were classified into two groups: (1) psoas and (2) core muscles. The two muscle groups were segmented to calculate low-attenuation muscle (LAM) area, this parameter being considered an index of fat infiltration in the skeletal muscle [25]. VAT and SAT areas were segmented using an attenuation range of −190 to −30 HU, whereas the LAM areas of core, psoas, and mid-thigh muscles were calculated using a range of 0 to 34 HU. This detailed method has already been published [26,27].

### 2.7. Cardiorespiratory Fitness

As previously described [28], CRF was assessed by a submaximal standardized exercise test on a TMX 425 treadmill (Trackmaster, Newton, KS, USA) connected to a QuarkB2 monitor (Cosmed, Rome, Italy). The test consisted of a 3 min warmup at 2.5 mph and 0% slope followed by an increase in speed and slope for three to four stages of 5 min in order to reach 70–80% of the age-predicted maximal heart rate. In these present analyses, the estimated metabolic equivalent of task (MET) reached at a target heart rate of 150 beats/minute was the fitness outcome retained for analyses [28,29].

### 2.8. Oral Glucose Tolerance Test

Following a 12 h overnight fast, a 75 g oral glucose tolerance test (OGTT) was performed. Blood samples were drawn at 0, 15, 30, 45, 60, 90, 120, 150, and 180 min to assess glucose and insulin concentrations. Plasma glucose levels were measured enzymatically while plasma insulin levels were measured with a radioimmunoassay [30,31]. The total glucose and insulin areas under the curve (AUC) during the OGTT were determined using the trapezoid method between 0 and 180 min. The homeostasis model assessment of insulin resistance (HOMA-IR) was calculated from fasting glucose and insulin levels [32].

### 2.9. Plasma Lipoprotein/Lipid Profile

Participants had to be in a fasting state for at least 12 h before blood sampling. Plasma cholesterol, low-density lipoprotein (LDL) cholesterol, HDL cholesterol, triglycerides, and apolipoprotein B and apolipoprotein A1 levels were determined using standardized procedures [33,34,35,36]. Triglyceride and cholesterol concentrations were determined in plasma and lipoprotein fractions using a Technicon RA-500 (Bayer Corporation, Tarrytown, NY, USA), and enzymatic reagents were obtained from Randox (Crumlin, UK). Triglyceride-rich lipoproteins were first removed via ultracentrifugation. The HDL fraction was obtained after precipitation of LDL in the infranatant (density ≥ 1.006 g/mL) with heparin and MnCl_2_. Triglyceride and cholesterol concentrations were determined before and after the precipitation step. Apolipoprotein B and A1 levels were obtained in plasma by nephelometry using polyclonal antibodies on the Behring BN ProSpec (Dade Behring, Marburg, Germany).

### 2.10. Diet Assessment

At baseline and years 1, 2, and 3, participants completed a 3-day dietary record, including a nonworking day to evaluate mean daily energy and macronutrient intake using Nutrifiq2001 software (version 2.0; Université Laval). The reported quantities and types of foods were reviewed by a nutritionist. Diet quality was calculated using the Dietary Approaches to Stop Hypertension (DASH)-derived diet quality score [37].

### 2.11. Physical Activity Level

At baseline and years 1, 2, and 3, participants were asked to complete the 3-day Bouchard Activity Record, which allowed for estimation in min/day of sedentary time as well as PA of light, moderate, and vigorous intensity [38]. PA sessions were self-reported by the participants and revised throughout the meetings with the kinesiologists during the 3 years of this study. This allowed for the calculation of the percentage of active weeks (at least one day with PA), the percentage of inactive weeks (complete inactivity during a week), and the percentage of forced rest for medical reasons during the intervention year and during the 2-year maintenance period. When participants self-reported a frequency associated with their PA but gave no information on the average duration of their PA session, it was assumed that they were active for 15 min. On the other hand, when they self-reported a certain duration of PA but gave no information on the frequency during the week, it was assumed that they performed one PA session. Finally, when participants declared being physically active but gave no information on the volume (frequency or duration), an assumption was made that they performed the same amount of PA as their last mean volume reported. As such, the mean annual PA volume was calculated for each year using the following formula: annual total PA sessions or minutes/(364 − active days with no reported volume). The mean volume of PA during active weeks was also calculated to see if participants met the prescribed PA recommendations when they declared being active.

### 2.12. Statistical Analyses

Results are expressed as mean ± SD in tables and as mean ± SEM in figures. An ANOVA for repeated measures was performed to compare participants throughout each phase of this study. Participants were then divided into two subgroups based on CRF changes observed during the maintenance period. Those whose CRF deteriorated more than 0.5 MET were classified in the CRF− group (*n* = 36), whereas participants whose CRF was improved (delta CRF ≥ 0.5 MET) or maintained (delta CRF between −0.5 and 0.5 MET) were classified in the CRF+ group (*n* = 36). An ANOVA for repeated measures was performed to compare changes in CMR markers during the maintenance period between CRF groups. Univariate Pearson’s correlations were computed to explore associations between changes in LAM areas and changes in CRF or HOMA-IR. A multivariable stepwise regression analysis was also performed with changes in VAT, core LAM areas, METs, PA min/week, DASH score, and energy intake as independent variables and changes in glucose at 120 min, glucose AUC, insulin AUC, and HOMA-IR as dependent variables. Statistical significance was set at *p* < 0.05. All statistical analyses were performed with SAS 9.4 version 15.1 (SAS Institute, Cary, NC, USA).

## 3. Results

Of the 144 participants initially involved, 117 completed the 1-year intervention (81% retention rate), and 94 completed the 3-year protocol (65% retention rate). Among those, 88 were included in these analyses as they had complete CT imaging data. With the exception of being older at baseline (49 years old vs. 45 years old; *p* < 0.05), the characteristics of the sample of 88 men included in the present analyses were not different from the 56 participants without complete 3-year CT imaging data.

Table 1 shows markers of PA and diet during the 3-year protocol. The mean number of steps/day increased from 7808 ± 2782 at baseline to 9682 ± 2966 after the intervention (*p* < 0.05) and was maintained during the 2-year maintenance period. Minutes per day of MVPA and vigorous-intensity PA (VPA) increased from 17.2 ± 41.5 and 3.0 ± 8.7 at baseline to 36.3 ± 50.3 and 17.3 ± 21.8 after the 1-year intervention (*p* < 0.05) and remained stable during the maintenance period. During the intervention year, participants reported being engaged in PA in 80.4 ± 19.4% of the weeks. Compliance decreased to 74.2 ± 23.3% at year 2 (*p* = NS), and further to 66.2 ± 26.4% at year 3 (*p* < 0.05 compared to year 1 and year 2). The percentage of inactive weeks during the intervention was 12.8 ± 15.8% and increased to 18.5 ± 22.1% at year 2 (*p* = NS) and to 21.9 ± 22.9% at year 3 (*p* < 0.05 compared to year 1). As a result, the mean annual PA sessions/week and mean annual PA min/week progressively decreased from 2.8 ± 1.4 sessions/week during the intervention to 2.2 ± 1.5 sessions/week at the end of this study (*p* < 0.05 compared to year 1) and from 144.5 ± 80.0 min/week to 107.1 ± 73.2 min/week (*p* < 0.05 compared to year 1), respectively. During active weeks, participants took part in at least 150 min/week throughout this study, meaning that despite a decrease in the annual PA volume, participants still achieved PA recommendations [39,40]. The reported total energy, protein, and lipid intake as well as overall diet quality score improved after the intervention (*p* < 0.05) and remained stable during the maintenance period.

As shown in Table 2, body weight, BMI, and waist circumference decreased following the intervention (*p* < 0.05) but increased during the maintenance period (*p* < 0.05) despite remaining under baseline values (*p* < 0.05). During the maintenance period, improvements in the lipoprotein/lipid profile remained significant (*p* < 0.05); only apolipoprotein A1 levels decreased (*p* < 0.05). Glucose at 120 min and glucose AUC significantly increased during the maintenance period (*p* < 0.05) to values that were no longer different from baseline. However, insulin response (AUC) remained stable during the 2-year maintenance period, whereas insulin resistance (HOMA-IR) significantly decreased (*p* < 0.05). Regarding CRF, despite a small decrease of 3.5% in METs during the maintenance period (*p* < 0.05), it remained significantly higher than the baseline values (*p* < 0.05).

Table 2 also presents the participants’ abdominal adiposity and IMF indices. VAT and SAT decreased following the 1-year intervention (*p* < 0.05), with a partial rebound observed during the maintenance period (*p* < 0.05). However, even though LAM areas for psoas, core, and mid-thigh muscles decreased during the intervention (*p* < 0.05), values all returned to baseline levels after the 2-year maintenance period.

**Figure 1:**
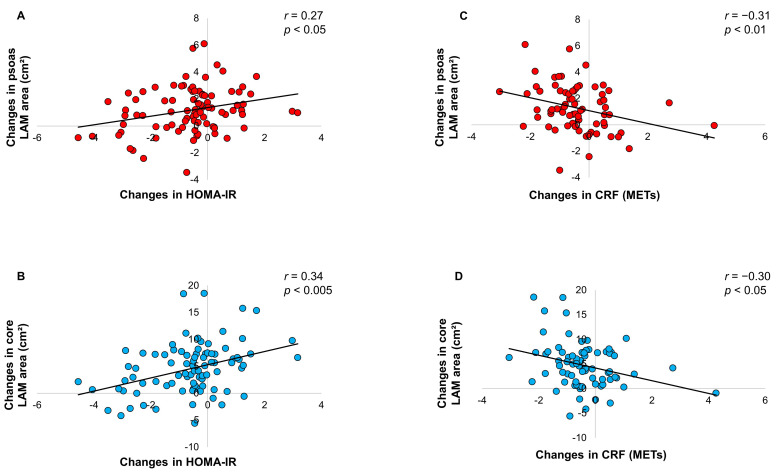
Changes in low-attenuation muscle (LAM) areas vs. changes in insulin resistance or cardiorespiratory fitness (CRF). Relationships between changes in LAM areas at the psoas and core and changes in homeostasis model assessment of insulin resistance (HOMA-IR) (panels (**A**,**B**)) or changes in metabolic equivalent of tasks (METs) (panels (**C**,**D**)) during the 2-year maintenance period. *n* = 88 participants for panels (**A**,**B**), and *n* = 72 participants for panels (**C**,**D**).

Changes in psoas and core LAM areas during the maintenance period were significantly correlated with changes in insulin resistance (Figure 1A,B), whereas mid-thigh LAM area changes did not correlate with changes in HOMA-IR (*r* = −0.16, *p* = NS).

Regarding CRF changes, complete data at year 1 and year 3 were available for 72 of the 88 participants. As shown in Figure 1C,D, significant negative correlations were observed between changes in CRF and changes in psoas and core LAM areas during the maintenance period, while no correlation was observed with changes in mid-thigh LAM area (*r* = −0.08, *p* = NS). The association between changes in CRF and changes in HOMA-IR did not reach significance (*r* = −0.20, *p =* NS).

Table 3 presents the characteristics of participants based on their CRF changes during the 2-year maintenance period (worsening: CRF− vs. maintenance/improvement: CRF+). During the 1-year intervention, neither group differed regarding adherence to PA and dietary recommendations (*p* = NS for changes in MVPA and energy intake). While both groups showed a rebound in the improvement of their CMR variables after the maintenance period, subjects who maintained or increased their CRF only regained half of their BMI (*p* < 0.005), waist circumference (*p* < 0.0005), VAT volume (*p* < 0.05), and psoas and mid-thigh LAM areas (*p* < 0.05) compared to those who worsened their CRF. During the maintenance period, the CRF+ group was also more physically active (MVPA) according to the Bouchard Activity Record (*p* < 0.05) and maintained their number of daily steps compared to the CRF− group (*p* < 0.05). No differences between the groups were observed regarding the lipid profile (*p* = NS for cholesterol, LDL cholesterol, HDL cholesterol and triglyceride levels).

**Figure 2:**
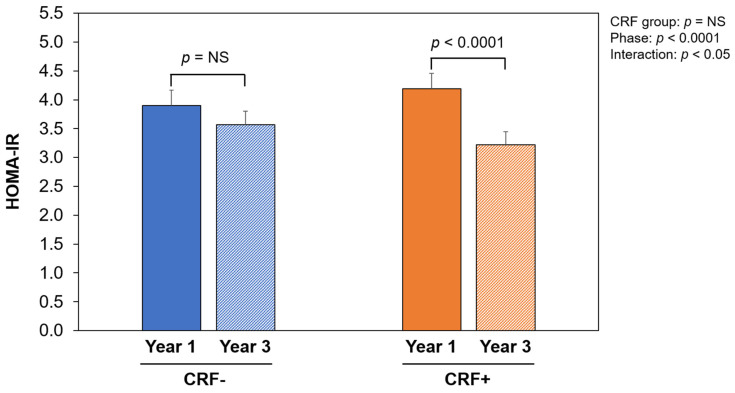
Changes in insulin resistance according to subgroups of cardiorespiratory fitness (CRF) changes. Homeostasis model assessment of insulin resistance (HOMA-IR) at year 1 and year 3 as a function of CRF response over the maintenance period (worsening: CRF− vs. maintenance/improvement: CRF+). NS, not significant.

Figure 2 shows changes in HOMA-IR according to subgroups of CRF changes. Improvements in the HOMA-IR were significantly greater in the CRF+ group compared to the CRF− group (*p* < 0.05).

Finally, to sort out which variables were associated with changes in glucose and insulin homeostasis during the 2-year maintenance period, multivariable stepwise regression models were computed. The results showed that changes in VAT volume partially explained the variation of glucose at 120 min, glucose AUC, and insulin AUC (Table 4). Changes in the core LAM area contributed to explaining some of the variation of insulin resistance, whereas changes in PA min/week only showed a significant contribution to changes in insulin resistance. Finally, the variation of insulin AUC was partially explained by changes in energy intake.

## 4. Discussion

The main objectives of the SYNERGIE study were (1) to document the long-term effects of a lifestyle intervention targeting PA and nutrition on the CMR profile of men with visceral obesity and (2) to examine whether long-term CMR profile changes were more closely related to changes in weight, VAT, or lifestyle habits (PA and nutrition). It is relevant to point out that SYNERGIE was not designed as a randomized controlled trial to evaluate the efficacy of a lifestyle intervention on the CMR profile as such interventions have already been shown to be effective [41,42,43]. The present analyses were conducted to explore how variations in IMF and CRF could explain the long-term improvement in insulin sensitivity in physically active men who regained VAT during a 2-year maintenance period that followed a 1-year lifestyle intervention.

### 4.1. Relative Deterioration in the CMR Profile

As already reported [16,37,44,45] and shown in Table 2, the 1-year lifestyle intervention significantly improved the CMR profile, as VAT and HOMA-IR were reduced by 30% and 19%, respectively, while CRF was increased by 20%. However, we observed a relative deterioration in some variables of the CMR profile during the 2-year maintenance period, as VAT increased by 22% while remaining lower than baseline values. This pattern of response with progressive drift and rebound over time was also observed in other lifestyle intervention studies, such as the Look AHEAD trial [46], the Finnish Diabetes Prevention Study [47], and the Diabetes Prevention Program [42]. Our results are concordant with these three studies as our participants lost 7.5% of their waist circumference and 8.3% of their body weight during the first year of the intervention, while showing regains of 3.8% and 3.6%, respectively, during the 2-year maintenance period.

### 4.2. Further Improvement in Insulin Resistance

Despite the increase in weight and VAT observed during the maintenance period, insulin resistance further improved. For instance, HOMA-IR decreased by 19% after the 1-year intervention and by a further 6% during the maintenance period. This result is similar to the Finnish Diabetes Prevention Study, where glycated hemoglobin slightly improved during years 2 and 3 of the study after an initial decrease over the first year [47]. In our study, changes in core LAM areas contributed to 11.4% of the long-term variation in HOMA-IR, suggesting an association between this ectopic fat depot and insulin resistance. While annual PA volume explained 6.8% of the variation in HOMA-IR, CRF did not come out as a significant factor in our multivariable regression model. Given that CRF is affected by the intensity of PA, we propose that participants might have been engaged in a sufficiently large volume of low-intensity PA to improve insulin sensitivity despite not having an influence on CRF. Taken together, these results suggest that lifestyle interventions involving regular PA could have long-term positive effects on insulin sensitivity despite some deteriorations in cardiometabolic variables, emphasizing the importance of promoting the adoption and adherence of a physically active lifestyle in at-risk patients.

### 4.3. Physical Activity and CRF during the Maintenance Period

Lifestyle interventions are known to increase PA in response to behavioral changes, but long-term adherence to new behaviors remains a challenge [48]. In this present study, steps/day and min/day of MVPA and VPA did not change during the maintenance period compared to the initial intervention year. Regarding self-reported PA, the mean annual volume of PA decreased during the maintenance period compared to the 1-year intervention. The challenge of sustaining lifestyle changes following the 1-year intervention suggests that preventive medicine approaches should include kinesiologists to optimally manage individuals with increased CMR. However, despite the global annual decrease in PA during the maintenance period, PA recommendations of 150 min of PA/week [39,40] were nevertheless met during self-declared active weeks, suggesting a partially sustained behavioral change. These results indicate that it could be possible to design lifestyle interventions targeting long-term maintenance of increased PA habits in clinical practice [49].

### 4.4. Increase in IMF

IMF is known to be elevated in sedentary individuals and in subjects with obesity, as well as being associated with insulin resistance [12,50]. In our study, our hypothesis that the improvement in insulin resistance observed during the maintenance period could be linked to a beneficial effect of PA/exercise on IMF mediated by changes in CRF was not confirmed by our findings. On the contrary, psoas, core, and mid-thigh LAM areas increased significantly during the 2-year maintenance period, with values no longer different from baseline levels. To the best of our knowledge, no studies have evaluated the long-term effects of a lifestyle intervention on IMF in sedentary individuals with visceral obesity. However, as discussed in two different reviews, lifestyle interventions including PA produce inconsistent changes [14,15]. This could be partly explained by the “athlete paradox”, where endurance-trained athletes have as much IMF as sedentary individuals with/without obesity. As opposed to sedentary individuals or patients with obesity, athletes have a high oxidative capacity to use this fat depot as an energy source during exercise [51]. The impaired oxidative capacity of sedentary individuals with obesity could result in the accumulation of metabolites such as diacylglycerols and ceramides, with negative consequences on indices of insulin sensitivity [15]. Thus, our study raises an important issue which remains to be addressed. Our 1-year intervention study resulted in a decrease in IMF as participants became physically active. Following the intervention, participants still achieved, most of the time, approximately 150 min of endurance exercise during the 2-year maintenance period with a further decrease in insulin resistance. It is therefore possible that the maintained level of PA simultaneously increased IMF levels and the muscle oxidative capacity, resulting in a further improvement in insulin sensitivity. This hypothesis is supported by a study by Dubé and colleagues [52] in which a 16-week exercise program conducted in sedentary subjects with overweight or obesity increased IMF by 21% while improving insulin sensitivity by 21% and decreasing diacylglycerol and ceramide levels. As we have no data on muscle oxidative capacity, further studies will be necessary to understand this paradox.

### 4.5. CRF Improvement during the Maintenance Period

Since the LAM area at the abdominal level correlated with insulin resistance and CRF, we also examined the impact of CRF changes during the maintenance period on LAM areas and HOMA-IR. Despite a slight deterioration in the CMR profile among men who maintained/improved or decreased their CRF, the CRF+ group only regained half of the changes in BMI, waist circumference, VAT volume, and psoas and thigh LAM areas compared to the CRF− group. The CRF+ group also significantly reduced their level of insulin resistance compared to the CRF− group. This finding could be potentially explained by the fact that an increased CRF is associated with lower VAT and liver fat, which could, in turn, lead to improved insulin sensitivity [53]. In addition, studies have shown that exercise training protocols that ameliorate CRF levels and reduce body fat and visceral fat could increase adiponectin concentrations [54]. Considering the key role played by adiponectin on systemic insulin sensitivity and glucose homeostasis [55], one cannot exclude the possibility that hormones such as adiponectin may also be involved in the improvement of insulin sensitivity. This remains to be explored.

Taken together, our results show that long-term maintenance/improvement in CRF could mitigate the attenuation of the beneficial effects of a lifestyle intervention, emphasizing the value of the long-term monitoring of CRF in clinical settings. Our results are in line with the mounting evidence showing that CRF is one of the best predictors of cardiometabolic and mortality outcomes [3,56]. It is therefore important to consider evaluating CRF in clinical settings, as adding this “lifestyle vital sign” to traditional risk factors improves long-term cardiovascular mortality assessment [57].

### 4.6. Strengths and Limitations

The major strength of our study is its longitudinal design, allowing a long-term (2 years) follow-up after a 1-year lifestyle intervention. Our findings help better understand the lasting effects of a lifestyle intervention on CMR and insulin resistance in men with visceral obesity. Another strength of this study was the use of CT, which allowed for precise measurement of VAT and SAT as well as an estimate of IMF using LAM areas. CRF was also measured using a submaximal exercise test. However, this study has some limitations. As we have previously documented major sex differences in body fat distribution, men being more prone to VAT accumulation than women [58,59], this present study was conducted in men only. Because of the high cardiometabolic risk associated with visceral obesity [60], there was a need to document the effects of a lifestyle intervention program in a group of men with high-risk visceral obesity. As menopause is associated with an acceleration in VAT deposition, similar lifestyle intervention studies will have to be conducted in post-menopausal women in order to examine the interaction with sex hormone levels, which obviously play a role in the redistribution of body fat during that period. Moreover, as no measures of ceramides, diacylglycerols, and the muscles’ oxidative capacity were performed, it was not possible to deepen the athlete’s paradox hypothesis. Another limitation of our study is that PA was self-reported through meetings with the kinesiologists and questionnaires, which could induce some recall bias [61]. Future use of accelerometers or smartwatches will be useful in order to better assess long-term PA patterns in response to lifestyle intervention and its impact on cardiometabolic health.

## 5. Conclusions

Following a 1-year lifestyle intervention that significantly improved CMR factors in sedentary men with visceral obesity, a regain in VAT was observed during a 2-year maintenance period as well as a slight deterioration in subjects’ global CMR profile. These present analyses document the independent contribution of IMF and PA on insulin sensitivity, emphasizing the importance of promoting regular PA among men with high CMR. The results also show that long-term improvement in CRF tends to offset/limit the deterioration in the CMR profile while even contributing to a further improvement in insulin sensitivity. These results are consistent with the notion that CRF is an important marker of health and should be monitored for the long-term management of patients with increased CMR.

## Figures and Tables

**Table 1:** Markers of physical activity and diet throughout each phase of the SYNERGIE study.

Variables	Baseline	Year 1	Year 2	Year 3
Mean annual consultations		15 ± 2	10 ± 2 ^b^	10 ± 3 ^b^
Physical activity level				
Pedometer (steps/day)	7808 ± 2782	9682 ± 2966 ^a^	9235 ± 2871 ^a^	9077 ± 2893 ^a^
MPA (min/day)	14.2 ± 41.5	19.0 ± 49.9	12.3 ± 29.4	10.7 ± 34.6
VPA (min/day)	3.0 ± 8.7	17.3 ± 21.8 ^a^	18.3 ± 29.4 ^a^	19.3 ± 34.3 ^a^
MVPA (min/day)	17.2 ± 41.5	36.3 ± 50.3 ^a^	30.6 ± 37.5 ^a^	30.0 ± 49.5 ^a^
Yearly physical activity				
% active weeks	-	80.4 ± 19.4	74.2 ± 23.3	66.2 ± 26.4 ^b,c^
% inactive weeks	-	12.8 ± 15.8	18.5 ± 22.1	21.9 ± 22.9 ^b^
% forced break *	-	1.8 ± 7.2	4.8 ± 9.2 ^b^	6.4 ± 14.5 ^b^
% unknown weeks	-	5.0 ± 11.1	2.5 ± 4.9	3.5 ± 6.9
Mean PA sessions/active week	-	3.4 ± 1.3	3.5 ± 1.8	3.3 ± 1.4
Mean PA min/active week	-	172.8 ± 79.2	154.8 ± 94.5	158.2 ± 82.8
Mean annual PA sessions/week	-	2.8 ± 1.4	2.5 ± 1.5	2.2 ± 1.5 ^b^
Mean annual PA min/week	-	144.5 ± 80.0	116.4 ± 90.8 ^b^	107.1 ± 73.2 ^b^
Energy intake and diet quality				
Total energy intake (kcal/day)	3026 ± 660	2518 ± 537 ^a^	2533 ± 547 ^a^	2427 ± 518 ^a^
Protein intake (% of energy intake)	16.2 ± 3.0	19.0 ± 2.7 ^a^	18.3 ± 3.5 ^a^	18.1 ± 3.0 ^a^
Fat intake (% of energy intake)	34.4 ± 6.1	30.5 ± 5.4 ^a^	31.0 ± 5.4 ^a^	31.2 ± 4.7 ^a^
Carbohydrate intake (% of energy intake)	45.1 ± 6.9	47.3 ± 6.1 ^a^	46.8 ± 6.4	47.1 ± 6.0
DASH-derived diet quality score	35.5 ± 10.4	52.0 ± 13.7 ^a^	-	50.0 ± 12.6 ^a^

Data are means ± SD; *p* value represents the result of an ANOVA for repeated measures: ^a^ Different from baseline, *p* < 0.05; ^b^ different from year 1, *p* < 0.05; ^c^ different from year 2, *p* < 0.05; * break for medical reasons; DASH, Dietary Approaches to Stop Hypertension; MPA, moderate-intensity physical activity; MVPA, moderate- to vigorous-intensity physical activity; PA, physical activity; VPA, vigorous-intensity physical activity.

**Table 2:** Participants’ anthropometric, cardiometabolic, and imaging characteristics throughout each phase of the SYNERGIE study.

Variables	Baseline	Year 1	Year 3	% Change Baseline—Year 1	% Change Year 1—Year 3
Age (years)	49.0 ± 8.2	50.1 ± 8.2	52.1 ± 8.2		
Anthropometry					
Body weight (kg)	93.9 ± 11.8	86.9 ± 12.1 ^a^	90.1 ± 12.1 ^a,b^	−7.5	3.8
Body mass index (kg/m^2^)	30.9 ± 3.1	28.6 ± 3.2 ^a^	29.5 ± 3.4 ^a,b^	−7.4	3.4
Waist circumference (cm)	107.8 ± 9.3	98.9 ± 10.2 ^a^	102.5 ± 10.0 ^a,b^	−8.3	3.6
Lipoprotein/lipid profile					
Total cholesterol (mmol/L)	5.11 ± 0.77	5.05 ± 0.72	5.03 ± 0.74	−0.1	0.6
LDL cholesterol (mmol/L)	3.09 ± 0.65	3.21 ± 0.69	3.13 ± 0.67	6.0	−0.7
HDL cholesterol (mmol/L)	0.96 ± 0.18	1.09 ± 0.20 ^a^	1.08 ± 0.23 ^a^	15.5	−0.4
Cholesterol/HDL cholesterol	5.45 ± 0.94	4.73 ± 0.85 ^a^	4.79 ± 0.96 ^a^	−12.5	2.0
Triglycerides (mmol/L)	2.49 ± 0.93	1.87 ± 0.69 ^a^	2.01 ± 0.84 ^a^	−21.6	12.2
Apolipoprotein B (g/L)	1.08 ± 0.17	1.04 ± 0.18 ^a^	1.02 ± 0.17 ^a^	−3.6	−0.6
Apolipoprotein A1 (g/L)	1.14 ± 0.15	1.31 ± 0.16 ^a^	1.24 ± 0.17 ^a,b^	15.6	−4.4
Plasma glucose-insulin homeostasis					
Glucose at 120 min (mmol/L)	7.7 ± 1.6	6.7 ± 1.7 ^a^	7.3 ± 1.8 ^b^	−11.3	12.8
AUC glucose (mmol/L × 180 min × 10^−2^)	14.6 ± 2.3	13.5 ± 2.3 ^a^	14.3 ± 2.1 ^b^	−7.4	8.2
AUC insulin (pmol/L × 180 min × 10^−3^)	172 ± 79	104 ± 54 ^a^	94 ± 50 ^a^	−34.1	−1.3
HOMA-IR	6.0 ± 3.1	4.0 ± 1.7 ^a^	3.5 ± 1.4 ^a,b^	−19.4	−5.9
Cardiorespiratory fitness					
Exercise output at 150 beats/min (METs)	7.6 ± 1.4	9.0 ± 1.6 ^a^	8.6 ± 1.6 ^a,b^	19.7	−3.5
Abdominal adipose tissue					
L2-L3 VAT (cm^2^)	308.3 ± 75.5	231.5 ± 88.4 ^a^	273.1 ± 85.6 ^a,b^	−25.5	23.2
L2-L3 SAT (cm^2^)	198.2 ± 76.5	156.2 ± 67.0 ^a^	179.1 ± 74.1 ^a,b^	−20.7	16.0
L4-L5 VAT (cm^2^)	253.8 ± 72.7	179.2 ± 79.6 ^a^	211.5 ± 86.0 ^a,b^	−30.2	21.9
L4-L5 SAT (cm^2^)	301.7 ± 95.3	242.6 ± 91.9 ^a^	273.3 ± 95.1 ^a,b^	−19.8	14.2
VAT volume (cm^3^)	1955 ± 490	1440 ± 572 ^a^	1682 ± 547 ^a,b^	−27.1	22.1
SAT volume (cm^3^)	1725 ± 600	1381 ± 529 ^a^	1575 ± 578 ^a,b^	−19.6	14.7
LAM areas					
Psoas (cm^2^)	7.8 ± 2.5	6.3 ± 2.5 ^a^	7.4 ± 2.6 ^b^	−19.4	24.6
Core (cm^2^)	42.9 ± 11.0	37.0 ± 11.2 ^a^	41.5 ± 11.4 ^b^	−14.1	13.9
Mid-thigh (cm^2^)	64.4 ± 16.0	58.4 ± 18.2 ^a^	64.2 ± 19.9 ^b^	−9.7	10.8

Data are means ± SD; *p* value represents the result of an ANOVA for repeated measures: ^a^ different from baseline, *p* < 0.05; ^b^ different from year 1, *p* < 0.05; AUC, area under the curve; HDL, high-density lipoprotein; HOMA-IR, homeostasis model assessment of insulin resistance; LAM, low-attenuation muscle; LDL, low-density lipoprotein; SAT, subcutaneous adipose tissue; VAT, visceral adipose tissue.

**Table 3:** Cardiometabolic risk profile and lifestyle behaviors in CRF subgroups during the maintenance period.

Variables	CRF− (*n* = 36)			CRF+ (*n* = 36)			Group *p* Value	Phase *p* Value	Interaction *p* Value
	Year 1	Year 3	Delta	Year 1	Year 3	Delta			
Anthropometry									
Body mass index (kg/m^2^)	28.7 ± 3.0	30.0 ± 3.5	1.3 ± 1.1 ^a^	28.2 ± 3.8	28.7 ± 3.6	0.6 ± 0.9 ^a^	NS	<0.0001	<0.005
Waist circumference (cm)	98.4 ± 9.9	103.4 ± 10.4	5.0 ± 3.7 ^a^	98.2 ± 11.3	100.2 ± 10.3	2.0 ± 3.1 ^a^	NS	<0.0001	<0.0005
Plasma glucose-insulin homeostasis									
Glucose at 120 min (mmol/L)	6.79 ± 1.82	7.15 ± 1.80	0.36 ± 1.63	6.59 ± 1.60	7.17 ± 1.79	0.58 ± 1.81	NS	<0.05	NS
AUC glucose (mmol/L × 180 min × 10^−2^)	14.3 ± 2.3	14.8 ± 2.2	0.6 ± 1.4	12.7 ± 2.5	13.5 ± 1.9	0.8 ± 1.2	<0.05	<0.0005	NS
AUC insulin (pmol/L × 180 min × 10^−3^)	119.5 ± 60.9	115.6 ± 62.2	−3.9 ± 51.0	100.6 ± 53.8	77.2 ± 31.4	−23.3 ± 43.5	<0.05	<0.05	NS
Computed tomography									
VAT volume (cm^3^)	1359 ± 490	1693 ± 543	334 ± 265 ^a^	1363 ± 482	1549 ± 487	186 ± 194 ^a^	NS	<0.0001	<0.05
Psoas LAM area (cm^2^)	5.5 ± 2.1	7.3 ± 2.4	1.8 ± 1.8 ^a^	6.6 ± 2.5 *	7.4 ± 2.7	0.8 ± 1.6 ^a^	NS	<0.0001	<0.05
Core LAM area (cm^2^)	35.1 ± 9.4	40.6 ± 10.7	5.5 ± 5.4	36.6 ± 11.6	40.5 ± 11.6	3.9 ± 3.7	NS	<0.0001	NS
Mid-thigh LAM area (cm^2^)	53.2 ± 13.7	61.0 ± 15.6	7.7 ± 6.5 ^a^	60.5 ± 19.8	64.6 ± 20.2	4.1 ± 7.0 ^a^	NS	<0.0001	<0.05
Physical activity									
Pedometer (steps/day)	10,553 ± 2479	9050 ± 2870	−1503 ± 1838 ^a^	9577 ± 3552	9320 ± 3132	−257 ± 2449	NS	<0.005	<0.05
Mean annual sessions/week	2.7 ± 1.3	1.9 ± 1.2	−0.8 ± 1.4	2.9 ± 1.4	2.5 ± 1.5	−0.5 ± 1.7	NS	<0.005	NS
Mean annual PA min/week	143.8 ± 72.0	88.5 ± 56.1	−55.3 ± 78.8	144.0 ± 75.6	117.2 ± 77.0	−26.8 ± 92.1	NS	<0.0005	NS
MPA (min/day)	23.1 ± 51.5	6.3 ± 15.1	−16.8 ± 48.8	9.2 ± 24.7	13.0 ± 52.9	3.8 ± 56.3	NS	NS	NS
VPA (min/day)	18.6 ± 24.1	15.0 ± 20.9	−3.6 ± 26.5	15.6 ± 20.2	28.2 ± 48.2	12.6 ± 52.6	NS	NS	NS
MVPA (min/day)	41.7 ± 53.1	21.3 ± 29.4	−20.4 ± 50.2	24.8 ± 27.2	41.2 ± 67.7	16.4 ± 75.6	NS	NS	<0.05
Cardiorespiratory fitness									
Exercise output at 150 beats/min (METs)	9.5 ± 1.5	8.3 ± 1.3	−1.2 ± 0.6 ^a^	8.6 ± 1.8 *	8.9 ± 1.8	0.4 ± 0.9 ^a^	NS	<0.0001	<0.0001
Energy intake and diet quality									
Total energy intake (kcal/day)	2608 ± 524	2368 ± 425	−240 ± 482	2540 ± 480	2483 ± 510	−56 ± 548	NS	<0.05	NS
DASH-derived diet quality score	53.2 ± 14.5	49.3 ± 13.9	−3.8 ± 13.5	53.3 ± 13.1	54.9 ± 9.8	1.7 ± 10.5	NS	NS	NS

Data are means ± SD; ^a^ delta between year 1 and year 3, *p* < 0.05 in each group; * difference between groups at the corresponding year, *p* < 0.05; AUC, area under the curve; CRF, cardiorespiratory fitness; DASH, Dietary Approaches to Stop Hypertension; HOMA-IR, homeostasis model assessment of insulin resistance; LAM, low-attenuation muscle; METs, metabolic equivalent of tasks; MPA, moderate-intensity physical activity; MVPA, moderate- to vigorous-intensity physical activity; NS, not significant; PA, physical activity; VAT, visceral adipose tissue; VPA, vigorous-intensity physical activity.

**Table 4:** Multivariable regression analysis showing the contribution of independent variables to changes in glucose-insulin homeostasis.

		Model			
Dependent Variables	Independent Variables	Total R² × 100	Partial R² × 100	*p*	Standardized β
Δ Glucose 120 min		5.6			
	Δ VAT volume		5.6	<0.05	0.23572
Δ AUC glucose		13.6			
	Δ VAT volume		9.4	<0.05	0.31338
	Δ Energy intake		-	NS	−0.20663
Δ AUC insulin		29.8			
	Δ VAT volume		18.8	<0.005	0.28388
	Δ Energy intake		6.2	<0.05	−0.26920
	Δ Core LAM area		-	NS	0.27179
Δ HOMA-IR		18.3			
	Δ Core LAM area		11.4	<0.05	0.32159
	Δ PA min/week		6.8	<0.05	−0.26190

AUC, area under the curve; HOMA-IR, homeostasis model assessment of insulin resistance; LAM, low-attenuation muscle; NS, not significant; PA, physical activity; VAT, visceral adipose tissue; - not included in the model due to lack of significance.

## Data Availability

The data presented are available upon request from the corresponding author. The data are not publicly available due to privacy restrictions.
